# Radiofrequency Ablation for Early Superficial Flat Esophageal Squamous Cell Neoplasia: A Comprehensive Review

**DOI:** 10.1155/2020/4152453

**Published:** 2020-05-14

**Authors:** Siyu Lei, Sachin Mulmi Shrestha, Ruihua Shi

**Affiliations:** ^1^School of Medicine, Southeast University, Nanjing, China; ^2^Department of Gastroenterology, Zhongda Hospital Affiliated to Southeast University, No. 87 Ding Jia Qiao, Nanjing, Jiangsu 210009, China

## Abstract

Esophageal squamous cell carcinoma comprises the majority of esophageal carcinoma in the Eastern Asia. The need of early detection of precancerous neoplastic lesions and cancer has been necessitated due to the probability of progression to the advanced stage and its poor prognosis. In recent times, many endoscopic modalities have come into practice for early detection and treatment. Endoscopic radiofrequency ablation (RFA) has been recommended as an efficient therapy in treating the dysplastic mucosa in Barrett's esophagus (BE). Its potential in reversing neoplastic lesions in squamous epithelium has been gradually explored. This article is aimed at reviewing the current evidence regarding the use of RFA on esophageal squamous cell neoplasia.

## 1. Introduction

Esophageal cancer is the seventh most common cancer in the world, with the mortality rate ranking sixth globally [1]. In China, esophageal cancer is the fourth leading cause of cancer death and the sixth most commonly diagnosed cancer [2]. Unlike in many Western countries where adenocarcinoma accounts for the majority of esophageal malignancy, esophageal squamous cell carcinoma predominates in esophageal cancer in China [3].

The precancerous lesion—squamous intraepithelial neoplasia is usually divided into three grades according to the depth of the dysplastic cell invading the epithelium, including low-grade intraepithelial neoplasia (LGIN, involving the lower third of the epithelium), moderate-grade intraepithelial neoplasia (MGIN, involving the lower two-thirds), and high-grade intraepithelial neoplasia (HGIN, involving the whole epithelium but not penetrating the basal layer) [4]. Early esophageal squamous cell neoplasia (ESCN), consisting of the above-mentioned squamous intraepithelial neoplasia and esophageal squamous cell carcinoma which confined to lamina propria mucosae (m_2_), is considered to have a very low risk of lymph node metastasis and lymphatic invasion, which rationalizes the endoscopic treatment for these lesions [5, 6].

Extensive researches have proved the efficacy of endoscopic treatment for early ESCN. Compared with esophagectomy, endoscopic therapy shows an equal curative effect, lower perioperative mortality, and less adverse events [7]. Endoscopic treatment can be tissue destructive or nondestructive according to the requirement for further histological analysis. The former includes radiofrequency ablation (RFA) and argon plasma coagulation (APC). The latter contains endoscopic mucosal resection (EMR) and endoscopic submucosal dissection (ESD). Nondestructive methods enable a thorough histological analysis of resected tissue and an accurate evaluation of the effectiveness of treatment. Nevertheless, to perform endoscopic resection (ER), especially ESD, is relatively risky and technically demanding considering the longer operating time and more chance of perforation and strictures compared to destructive methods. It is worth noting that RFA may have an advantage in treating larger lesions and causing less strictures than ER [8]. Since RFA has been recommended as a preferred endoscopic therapy for Barrett's esophagus (BE) in recent years [9], there is increasing interest in its application in ESCN.

This review is aimed at discussing the state of the art knowledge on the application of RFA on esophageal squamous cell neoplasia and the current controversies on this topic. We retrieved PubMed and Web of Science databases from inception to March 2020 using MeSH, text words, and thesaurus including but not limited to esophageal neoplasms, squamous cell neoplasm, esophagoscopy, and radiofrequency catheter ablation. All articles retrieved were artificially reviewed through title and abstract to filtrate relevant ones. The reference list of some articles was also looked up in order to reduce possible omission. The term “RFA” would represent “balloon-based RFA” in the content unless otherwise noted.

### 1.1. Preclinical Exploration of RFA on Esophageal Squamous Epithelium

In 2004, Ganz et al. first evaluated varying energy density and power of balloon-based bipolar radiofrequency electrode system in the porcine and human esophagus in a four-phase trial [10]. In the first phase, all swine were euthanized immediately after being ablated at varying energy density and power. Complete eradication of epithelium occurred when energy density was above 9.7 J/cm^2^. In the second phase, the swine died or were euthanized in 2 to 4 weeks after ablation, and the rate and severity of esophageal stricture were reported to be positively correlated with energy density; with low energy density (9.7 and 10.6 J/cm^2^), no stricture was seen and with high energy density (>22 J/cm^2^), stricture was seen in all animals. In the third phase, the depth of ablation was evaluated histologically, and the results demonstrated a positive correlation between the energy density and the ablation depth, with the maximum ablation depth being the muscularis mucosae (m_3_) and superficial submucosa when energy density was set to 10 J/cm^2^ and 12 J/cm^2^, respectively. In the fourth phase, three patients with adenocarcinoma were enrolled. Radiofrequency ablation was performed 5 cm proximal to the tumor at 10 J/cm^2^ to 12 J/cm^2^. As a result, all patients achieved complete ablation of squamous epithelium and the ablation injury was confined to muscularis mucosae.

Dunkin et al. then performed balloon-based RFA on nonneoplastic esophageal squamous mucosa on human and concluded that a second application of ablation supplemented the first one without deepening the tissue injury [11]. They reported that the ideal regimens—10 J/cm^2^ (2×) or 12 J/cm^2^ (1× or 2×)—guaranteed complete elimination of esophageal epithelium and exempted the submucosa injury (the maximum ablation depth being muscularis mucosae).

Although the above preclinical studies were intended to deal with the esophageal intestinal metaplasia, the nature of using squamous epithelium as a substitution of intestinal metaplasia happened to imply its possible prospect in squamous epithelium lesions.

### 1.2. Indications of RFA for ESCN

The use of RFA on ESCN is most commonly restricted to Lugol's chromoscopy-verified unstained lesions (USLs) that histologically reveals MGIN, HGIN, or esophageal squamous cell carcinoma (ESCC) confined to m_2_. Additionally, the USLs should be completely flat, namely, types 0-IIb according to the Paris Classification of Superficial Neoplastic Lesions in the Digestive Tract. Moreover, the endoscopic ultrasound (EUS) is required to exclude submucosal invasion and lymphadenopathy, and computed tomography (CT) of the chest and upper abdomen is required to exclude the metastasis and lymphadenopathy. The rationales of the threshold of m_2_ lie in the following: first, the maximum ablation depth of RFA at 12 J/cm^2^ is up to the muscularis mucosae and 1000 *μ*m on the esophagus [10–13]. Theoretically, only lesions restricted to m_3_ are most likely to be completely eradicated. Second, the lesions confined to m_2_ show the lowest probability of lymph node metastasis (2%), and 69% of type 0-IIb lesions will not invade beyond m_2_ [14, 15]. Thus, the evaluation of invasion depth of tumor is a crucial part of eligibility assessment.

Pretreatment mucosa biopsies serve as the only histological evidence which directly shows the depth of lesion. However, the discrepancy between pretreatment biopsies and ER specimens was reported. Shimizu et al. enrolled 51 patients who were diagnosed with HGIN by biopsy specimens obtained from USLs. All of these patients underwent EMR afterwards, and lamina propria invasion and muscularis mucosae invasion are displayed in 12 (23.5%) and 4 (7.8%) of the lesions, respectively, in the EMR specimens [16]. Park et al. retrospectively analyzed 84 specimens of endoscopically resected superficial ESCN. Compared to the biopsy findings, 29 (34.5%) lesions showed discrepant result, of which 21 (72.4%) lesions upgraded from HGIN to ESCC [17]. Wang et al. compared the pre-ESD biopsies of esophageal squamous USLs with corresponding ESD specimens and found that 29.8% of the specimens had more advanced staging than originally believed [8]. Thota et al. reported similar results in the context of BE, with only 50% consistency between EMR histology and biopsy findings, and 21% of the lesions were underrated by pretreatment biopsies [18]. The discordance between biopsy and ER specimens could result from the inadequacy of the squamous biopsy specimens obtained that contain sufficient lamina propria [19, 20], the lateral extension nature of squamous carcinoma cell enabling it to escape from superficial biopsies [21], and the randomness of not sampling the most advanced region. Whether the application of jumbo biopsy forceps could improve the biopsy specimen adequacy in BE has been studied, but the results seem to be controversial [20, 22–25]. In esophageal squamous neoplasia, few studies have been established on the biopsy adequacy of jumbo biopsy forceps. In brief, the result of the biopsy may not be as reliable as it looks, which calls upon a combined methods to determine the tumor invasion depth.

New endoscopic imaging technology may provide a solution to this problem. The changes of intrapapillary capillary loops (IPCLs) observed under the magnifying endoscopy with narrow-band imaging (ME-NBI) correlate closely with the depth of superficial ESCC. There are mainly three classifications of IPCL changes: Inoue's, Arima's, and the new Japan Esophageal Society (JES) classifications. Wang et al. applied Inoue's classification of IPCLs (type IV and V1) to assist the selection of eligible patients for RFA treatment [26]. Although 20% of the patients developed local recurrence after successful RFA, no evidence was raised for the inaccuracy of pretreatment histological evaluation to be blamed. The new, simplified Japan Esophageal Society (JES) classification of IPCLs has proved to be high in accuracy for the diagnosis of the invasion depth of superficial ESCC, with 90.5% overall accuracy rate of the type B microvessels (representing cancerous lesions) [27] and satisfactory intra- and interobserver agreement [28]. One comparative study revealed higher specificity and comparable sensitivity of the new classification than Inoue's [29]. Considering the accuracy and simplicity of the new classification of identifying tumor invasion depth, it could be a promising tool for a more rigorous selection of the candidates for RFA for ESCN. Further trials need to be conducted under this presumption.

In addition, the second-generation optical coherence tomography (OCT) technology, also known as volumetric laser endomicroscopy (VLE), has established itself as a valid pretreatment method to assess the stage of suspected lesion. Trindade et al. compared high-frequency endoscopic ultrasound (HF-EUS) and VLE in pretherapy staging of superficial ESCC [30]. As a result, VLE showed higher accuracy in differentiating epithelium, lamina propria, and muscularis mucosa invasion in squamous cell carcinoma. This would be an advantage in deciding whether a patient is eligible to RFA.

Recently, the application of artificial intelligence (AI), especially deep learning technology, in endoscopic diagnosis holds strong appeal to endoscopists. An AI diagnostic system has shown higher accuracy in differentiating invasion depth of superficial ESCC than human [31]. With further development of AI-assisted diagnosis, more precise diagnosis will be in sight.

All in all, multiple modalities would be helpful for an accurate pretreatment staging of suspected lesions, yet the specific inclusion criteria still need to be polished according to the clinical suggestion.

### 1.3. Regimen of RFA

#### 1.3.1. Selection of Appropriate Device

For ESCN, a combined use of Barrx™ 360 RFA balloon catheter and focal RFA catheter such as Barrx™ 90 or 60 RFA focal catheter is the most common practice. For the Barrx™ 360 RFA system, two separate procedures are needed: sizing and ablating. A soft sizing balloon catheter of 4 cm long is first used to measure the esophageal inner diameter (EID) along the intended treatment area. The smallest measured EID will determine the size of the ablation catheter. Subsequently, the ablating catheter of one of the 5 specifications (18, 22, 25, 28, and 31 mm) is chosen to ablate the whole targeted area without sizing change. The shortcomings of this system are the multiple introductions of different devices and the fixed size of ablation catheter during the process.

Recently, a new Barrx™ 360 express RFA balloon catheter comes into being, aimed at overcoming the above shortcomings [32]. Barrx™ 360 express incorporates the sizing and ablating catheter to a self-adjusting balloon catheter with a 4 cm electrode array which could automatically inflate to a suitable size and ablate immediately after sizing without a separate introduction of ablation catheter. This new catheter intends to streamline the RFA procedure and shorten the operating time. Belghazi et al. initiated the pilot trial of applying the new system to BE within a standard 12 J/cm^2^–clean–12 J/cm^2^ regimen [32]. As a result, shorter procedure time and comparable efficacy were observed, but an unexpected high rate of esophageal scarring implied the aggressiveness of the standard regimen under this system. The authors thus suggested that a reduction in RFA energy density to 10 J/cm^2^ may counteract the increased risk of stricture result from the optimized contact between the electrode and the esophageal wall. Under this hypothesis, they further conducted a randomized clinical trial comparing different regimens using a 360 express RFA catheter [33]. The results revealed that although single application (1 × 10 J/cm^2^) of ablation may reduce the procedure time, it was not recommended in view of its poor BE regression response at 3 months compared to the standard group (2 × 10 J/cm^2^+cleaning). In addition, the simple-double regimen (2 × 10 J/cm^2^) caused a 21% stenosis rate, which went far beyond the common rate and hence was halted halfway. All the results above came from studies based on BE. There have been no studies on ESCN using the 360 express RFA system by now, yet a salutary lesson could be learned based on some known features of esophageal squamous epithelium to optimize the application of the 360 express RFA system in ESCN. First, the esophageal squamous epithelium is easier to be ablated than Barrett's epithelium [34], so the energy density control should be more cautious and conservative to avoid esophageal stenosis. Second, a higher post-RFA stenosis rate of ambiguous reasons has been reported in ESCN than BE [35], so the preventative measures should be taken into account before using 360 express RFA on ESCN.

Focal RFA catheters include the Barrx™ 90 RFA focal catheter and 60 RFA focal catheter which could be mounted on the tip of an endoscope and deliver energy to a small piece of tissue through the electrode array. They were designed for relative focal lesions especially the residual or recurrent lesions detected during follow-up endoscopy.

Another RFA catheter named “Barrx™ Channel RFA endoscopic catheter” has similar function as focal catheters but could pass through the working channel of endoscope so that it fits in some cases where tortuous and stenotic esophageal lumen hinders the introduction of focal RFA catheters [36].

### 1.4. RFA Protocol

**Figure 1:**
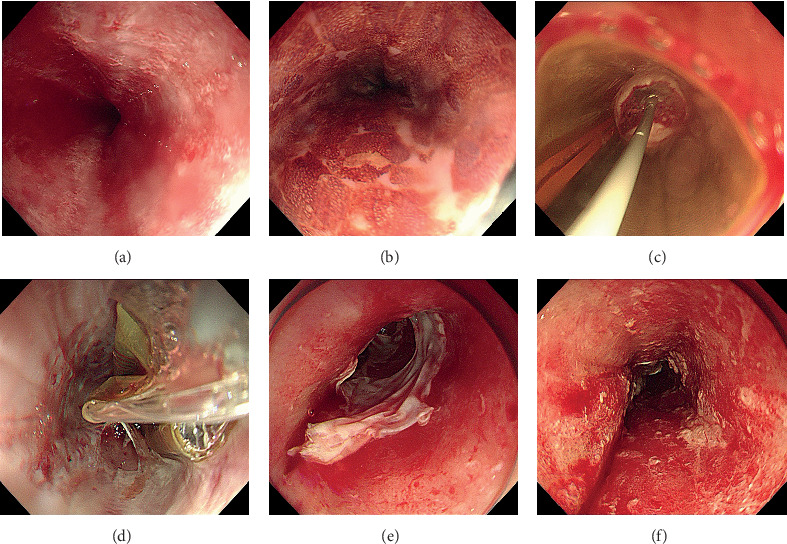
Circumferential balloon-based radiofrequency ablation of early squamous cell neoplasia. (a) White-light endoscopy showed rough and reddish areas. (b) Lugol's chromoendoscopy demonstrated multifocal unstained lesions. (c) Barrx™ 360 RFA balloon catheter placed in the esophagus before (d) and after the first circumferential ablation pass. (e) Appearance of the mucosa after the first circumferential ablation pass. (f) Appearance of the mucosa after cleaning the coagulum of treatment area.

There still has not been a “standard protocol” with regard to RFA for ESCN. The debate of using the appropriate energy density and the number of applications still exists among the endoscopists. As mentioned before, an energy density of 10 J/cm^2^ (2×) or 12 J/cm^2^ (1× or 2×) was the optimal treatment parameter for the ablation of human esophageal squamous epithelium. It was reported that two applications were beneficial to achieve complete remission and would not double the depth of injury [11]. The effect of RFA under these energy settings would reach muscularis mucosae but preserve submucosal tissue. Besides, Sharma et al. found that the sloughed epithelial would otherwise hamper the delivery of ablation energy if not cleaned before the second application when ablating nondysplastic BE [37]. Hence, two applications of 12 J/cm^2^ with intermediate cleaning (2 × 12 J/cm^2^+cleaning) have been thus far the most accepted regimen in the practice of circumferential RFA on BE. Accordingly, this regimen has now been a relatively common practice for RFA on ESCN (Figure 1).

The exploration of the optimal ablation regimen is still on. As for circumferential RFA, in terms of the energy density, except for 12 J/cm^2^, 10 J/cm^2^ was also applied in some studies, but unfortunately, the 10 J/cm^2^ regimens were not categorized as an independent group, which could not be used to analyze the dose effect. In terms of the appropriate number of applications, one of the most controversial issues is the use of single application regimen, which can be a great way to shorten the procedure time. Vilsteren et al. and Becker et al. applied single application of 12 J/cm^2^ in 6 patients, respectively [34, 38]. Eleven of these patients (Vilsteren 5/6, Becker 6/6) had received previous ER for nonflat lesions. All of them maintained complete remission (CR) during a follow-up of more than 10 months. He et al. reported that a single application of 12 J/cm^2^ without cleaning in a regimen group of 17 patients had a similar 12-month CR rate (82%), the lowest stricture rate (6%), and the least amount of procedure time than other groups which shared the double applications in common [39]. Of note, none of the patients had ER before. No differences were found among the groups regarding the rate of long-term CR or adverse events during a follow-up of 5 years [40]. They hence recommended that the single application may serve as a simple and efficient regimen. On the contrary, Haidry et al. reported a local recurrence rate of 50% (10/20) in a cohort of single application of 12 J/cm^2^ during a 12-month follow-up [41]. Although 5 of the 20 patients had previous ER, no detailed information was given about the previous ER rate of the patients with local recurrence. Even though the power of the results from the above studies was restricted by limited sample size, it seemed from the above first two studies that single application regimen was efficient when ER was performed in advance to eradicate the probably more advanced lesions. However, the study of Haidry et al. was not detailed enough to further verify this conjecture. Furthermore, He et al.'s study did not include patients with previous ER and still got justifiable long-term CR rate. Therefore, it is necessary to conduct more large-scale studies in the future to demonstrate the efficacy of single-application regimen and find out whether its efficacy has correlation with previous ER.

As for focal RFA, four applications of 15 J/cm^2^ with intermediate cleaning were reported to be too aggressive for ESCN. At present, one to three applications of focal RFA at 12 J/cm^2^ has been widely used. No trials comparing these different regimens are available till now.

### 1.5. Efficacy of RFA

Extensive studies have proved that RFA is safe and effective for eradication of Barrett's mucosa with the complete eradication rate of dysplasia and intestinal metaplasia being 91% and 78%, respectively [42]. There have been increasing but still limited studies to date focusing on the efficacy of RFA for treating ESCN. The characteristics of these studies are displayed in Table 1, and the outcomes are displayed in Table 2.

#### 1.5.1. Short-Term Outcome

Most of the existing studies illustrated the CR rate of RFA on ESCN within 12 months (6-12 months), which is herein defined as short-term studies [8, 26, 34, 35, 38, 39, 41, 43–46]. The CR rate of these short-term studies ranges from 50% to 100%. The overall CR rate would be higher than 84% irrespective of the study of Haidry et al., which had the lowest CR rate of 50%. The local recurrence rate was 0-50% (Table 2). The difference in the CR rate was conspicuous. Factors related to the discrepancy of the outcome as well as the risk factors of local recurrence will be discussed later.

#### 1.5.2. Long-Term Outcome

The only long-term study at present is a continuation study of one of the above short-term trials [40]. The patients of the initial study were continued to be followed up for 5 years, and 86% (67/78) of those patients who achieved complete remission at 12 months had a persistent effect at the end of the follow-up. Nine percent (7/78) and 5% (4/78) of patients had local recurrence and progression, respectively (Table 2).

#### 1.5.3. Factors Related to the Efficacy of RFA on ESCN

After careful review of the clinical trials above, several factors were suspected to affect the local recurrence or CR rate.

The first and most mentioned one was the pretreatment biopsy histology. Wang et al. reported that pretreatment biopsy results could independently predict the local recurrence of ESCN with the hazard ratio of ESCC to HIGN being 12.46 (95% confidence interval 1.12-138.44) [26]. He et al. found that a higher 12-month CR rate was associated with lower baseline histology grade [39]. As was mentioned before, the histological results of pretreatment biopsy may share less-than-ideal consistency with that of specimens obtained from ER, so whether this may act as a confounding factor that affects the local recurrence rate still needs to be clarified. Interestingly, a study reported that, in 17 superficial ESCC cases which were considered eligible for RFA by endoscopic image review, thirty-five percent were histologically underrated [47]. Although the estimation of the eligibility was mainly based on retrospective review of white light imaging and NBI without referring to prior biopsy, the use of RFA for ESCC should still be cautious or even restricted. In a word, researchers should take it seriously in deciding whether to enroll patients having ESCC in the future trials of RFA for ESCN.

The second factor identified to influence the outcome was the pink-color sign. Shimizu et al. first defined the pink-color sign as the light-pink part appeared in the iodine-unstained area during the fading of iodine brown in other places [48]. The esophageal squamous mucosa showing the pink-color sign had a high possibility (91.9%) to contain HIGN and ESCC. Yu et al. found out that the pink-color sign predicted 58% of the treatment failure at 12 months [40]. Twenty-seven percent of the patients who achieved CR at 12 months but developed local recurrence or progression and afterwards had pink-color sign. Therefore, the pink-color sign was recommended as the contraindication of RFA for ESCN by Yu et al. and further investigation on its diagnostic value is underway.

Third, ductal involvement (DI) or glandular involvement (GI) was not rare in esophageal squamous cell neoplasia. The prevalence of DI in esophageal squamous cell mucosal carcinoma was 11.9%-23.5% [49]. Although it was reported that DI or GI might not cause lymph node metastasis nor affect long-term prognosis of ESCN after ER, things could be different in the case of RFA. Wang et al. developed a cohort that analyzed the recurrent lesions after successful RFA treatment by acquiring the specimens through endoscopic resection [26]. As a result, eighty-six percent of the resected recurrent lesions had DI extending to the m_3_ or submucosal layer. The DI was located in the center of the recurrent lesion and showed similar reaction to the molecular marks as esophageal epithelial neoplastic cells did. As RFA may destroy the mucosa but reserve the submucosal layer, it is possible that these “submucosal DI” being the nest for tumor to come back. Therefore, ER especially ESD should be the optimal choice for the local recurrence of RFA to eliminate residual DI and GI. Lesions suspected of DI or GI should be cautious when applying RFA as the only therapy.

Other factors that had influence on the outcome of RFA for ESCN included the length of USLs, the number of the application of ablation, previous ER or not, and the experience and profession of the endoscopists.

### 1.6. Safety of RFA

#### 1.6.1. Esophageal Stricture

The most common adverse event was esophageal stricture even though the incidence of stricture after RFA for ESCN is relatively low compared to that of ER.

Current researches demonstrated that RFA caused approximately 0-28.6% postprocedure esophageal stenosis and all of which could relieve through a median of 2.5-5.5 sessions of dilation (Table 2). Compared to the stricture rate of other endoscopic methods which also result in a wide range of mucosa defect (e.g., ESD involving more than 3/4 of circumferential esophagus generating almost 100% stricture), the stricture rate of RFA seemed to be relatively low and trivial. However, the stricture rate of RFA for ESCN was higher than that of RFA on BE (0-9%) [35]. The underlying mechanism was not yet illustrated.

On the basis of the current clinical findings, the confirmed risk factor of RFA-related stenosis is the longitudinal tumor length, with a cut-off of 9 cm. Wang et al. applied the factor of longitudinal tumor length over 9 cm to the prediction of the post-RFA stricture rate of an exogenous ESCN cohort with 21 patients [46]. The results demonstrated a sensitivity and specificity of 80% and 81.2%, respectively. In addition, a previous history of ER and higher ablation energy density applied are also suspected to be associated with stenosis [34]. An animal study had revealed that Lugol's staining right before RFA caused more postprocedure stenosis, which may be due to the severe inflammation and fibrosis caused by Lugol's solution [50]. However, a clinical trial found an intriguing higher stricture rate in the group without Lugol's staining than in other groups with Lugol's staining [39]. So the influence of Lugol's solution may need further exploration.

The RFA-related strictures are reversible through esophageal dilation. The prevention methods of RFA-related stenosis are barely reported to date. Based on the past experience on the prevention of esophageal stricture after ER, local or systemic corticosteroids, preventive dilation, esophageal stent, and autologous cell sheets may play a role in the prevention of RFA-related stenosis.

#### 1.6.2. Other Adverse Events

Radiofrequency ablation was reported to be safely applied on patients with varices, which manifested its security and feasibility [43]. The mean incidence of bleeding (most being mucosal or submucosal hematoma) and laceration is 6.1% ± 0.08% and 4.2% ± 0.05%, respectively. Both of bleeding and laceration could relieve spontaneously or through simple symptomatic treatments. Perforation was reported in one case after dilation of a stenosis, and no perforation directly resulted from RFA was reported [34].

### 1.7. Combination of RFA and ER

Although ER has always been the optimal choice for ESCN, a high rate of stricture and adverse events has restricted its use on large lesions. One retrospective study comparing ESD and RFA including 47 ESD and 18 RFA patients demonstrated no difference in the treatment efficiency between the two [8]. But ESD consumed longer procedure time and led to more severe strictures than RFA did. A recent study demonstrated that even prophylactic steroid administration was applied before performing ESD in patients with circumferential ESCN, thirty-eight percent and 12% patients had refractory and unimproved strictures, respectively [51]. The combination of RFA and ER seems to be one possible way out of this dilemma. A large ESCN usually includes different grades of dysplastic lesions. For the endoscopically advanced part of a lesion, ER should be performed to get the part of the tumor which has the most advanced histological results to determine whether this lesion is still eligible for endoscopic therapy. If so, circumferential RFA could be applied subsequently to eradicate the residually less advanced mucosal dysplasia and possible synchronous and multifocal lesions.

Becker et al. combined multiple therapies including RFA, ESD, and EMR in the treatment of multifocal or recurrent superficial ESCC in 6 patients [38]. Complete remission was observed in all patients without adverse events nor local recurrence during a median of 10.5-month (IQR 10-13) follow-up. Vilsteren et al. conducted ER 2 months before RFA in 9 patients (69%) to eliminate visible or nonflat ESCN [34]. After RFA, all of the patients received complete remission and no local recurrence was detected during a median follow-up of 17 (IQR 11-22) months. In this study, one patient developed stricture after ER which impeded the use of circumferential RFA and thus been treated with focal RFA with multiple overlapping using an aggressive regimen (2 × 15 J/cm^2^+cleaning), which resulted in esophageal perforation. Apart from this, another two strictures and three mild adverse events during follow-up were managed with appropriate treatments. Besides, several studies had reported the successful application of ER or other endoscopic therapies (e.g., argon plasma coagulation (APC)) after successful RFA [8, 26, 34, 38, 41, 43]. All the above indicated that the combination of RFA and other endoscopic therapies was feasible and efficient. Radiofrequency ablation may not affect subsequent endoscopic therapies while it could be hampered by the stricture caused by previous ER. It is worth noting that in one study researchers performed RFA immediately after ER on 8 porcine models, one delayed perforation occurred, and three transmural inflammation and fibrosis of the muscularis propria were observed [52]. Although the porcine esophageal wall seemed thinner than humans', which may result in much deeper injury, the use of RFA immediately after ER should be avoided.

The attempt of combining RFA with ER for ESCN was encouraging. More large-sampled trials are needed to demonstrate the joint effect and optimal treatment paradigm of the multimodal therapy.

## 2. Outlook

The use of RFA for ESCN has showed initial success. Novel diagnostic tools and strategies such as ME-NBI, VLE, new JES classification of IPCL, and AI-assisted diagnosis are paving our way to more accurate pretreatment staging. With the ongoing clinical trials, the inclusion criteria and practical treatment regimens have been continuously refining in order to improve the outcomes. With stricture being the most common adverse event of RFA on ESCN, the probable mechanism behind remains to be elucidated. It is conceivable that the combination of RFA and other endoscopic therapies would benefit each other and have a wide prospect. As evidence on this topic accumulates, we have reason to believe that the answers to some of the controversial questions and the combined advantages will be clear in the future.

## Figures and Tables

**Table 1:** Characteristics of studies on RFA for ESCN.

First author	Year	Study design	Sample size	Inclusion criteria	Initial circumferential RFA regimen	Surveillance focal RFA regimen and other endoscopic modalities	Previous ER (%)	Follow-up regimen	Follow-up, mean or median (IQR) (m)
Pouw [44]	2008	Case report	1	HGIN/ESCC	2 × 12 J/cm^2^+cleaning	No	No	D3, M2, M4	4
Becker [38]	2011	Case series	6	ESCC	1 × 12 J/cm^2^	1 × 15 J/cm^2^	Yes (67%)	M1, M3, M6, and biannually thereafter	10.5 (10-13)
Vilsteren [34]	2011	Prospective case series	13	HGIN/ESCC	2 × 12 J/cm^2^+cleaning (1 × 12 J/cm^2^ in 6 cases)	4 × 15 J/cm^2^+cleaning between each two^†^	Yes (69%)	M3, M6, and annually thereafter	17 (11-22)
Bergman [35]	2011	Prospective cohort	29	MGIN/HGIN/ESCC	2 × 10 J/cm^2^ or 2 × 12 J/cm^2^±cleaning	3 × 12 J/cm^2^	No	M3, M6, M9, and M12	12
Haidry [41]	2013	Prospective cohort	20	HGIN/ESCC	1 × 12 J/cm^2^	1 × 12 J/cm^2^	Yes (25%)	M3, M6, M9, and M12	24 (17-54)
Wang [45]	2014	Prospective case series	7	HGIN/ESCC	2 × 12 J/cm^2^+cleaning	2 × 12 J/cm^2^+APC	No	M1, M3, M6, and biannually thereafter	10.5
Wang [8]	2015	Retrospective cohort	18	HGIN/ESCC	2 × 12 J/cm^2^+cleaning	2 × 12 J/cm^2^+APC	No	M1, M3, M6, and biannually thereafter	13.1
He [39]	2015	Prospective cohort	96	MGIN/HGIN/ESCC	1 × 12 J/cm^2^ or 2 × 10 J/cm^2^ or 2 × 12 J/cm^2^±cleaning	3 × 12 J/cm^2^	No	M3, M6, M9 and M12	12
Wang [46]	2016	Prospective cohort	30	HGIN/ESCC	2 × 12 J/cm^2^+cleaning	2 × 12 J/cm^2^+APC	No	M1, M3, M6, and biannually thereafter	17
Wang [43]	2017	Retrospective cohort	8	HGIN/ESCC	2 × 12 J/cm^2^±cleaning	3 × 12 J/cm^2^+APC	No	M1, M3, M6, and biannually thereafter	21.6
Wang [26]	2018	Prospective cohort	35	HGIN/ESCC	2 × 12 J/cm^2^+cleaning	2 × 12 J/cm^2^	No	M1, M3, M6, and biannually thereafter	40.1 (24-66)
Yu [40]	2019	Prospective cohort	78	MGIN/HGIN/ESCC	1 × 12 J/cm^2^ or 2 × 10 J/cm^2^ or 2 × 12 J/cm^2^±cleaning	3 × 12 J/cm^2^	No	M3, M6, M9, M12, and every 3 months or annually thereafter	60

RFA: radiofrequency ablation; ESCN: esophageal squamous cell neoplasia; ER: endoscopic resection; IQR: interquartile range; ESCC: esophageal squamous cell carcinoma; HGIN: high-grade intraepithelial neoplasia; MGIN: moderate-grade intraepithelial neoplasia; APC: argon plasma coagulation. ^†^Regimen adjustment was made to two applications of 15 J/cm^2^ with intermediate cleaning or three applications of 12 J/cm^2^ without cleaning.

**Table 2:** Outcomes of RFA for ESCN.

First author	Year	Mean or median (IQR) number of RFA	CR rate	Defined CR duration (m)	Recurrence (%)	Complications				
						Stricture rate	Mean or median (IQR) number of dilation	Perforation	Bleeding/hematoma	Laceration
Pouw [44]	2008	1	100%	4	0	0	—	0	0	0
Becker [38]	2011	1.7	100%	10^†^	0	0	—	0	0	0
Vilsteren [34]	2011	2 (1-3)	100%	2 months after last treatment	0	23%	3 (1-12)	1	1	2
Bergman [35]	2011	1.7	97%	12	1 (3%)	14%	2.5 (2-4)	0	0	1
Haidry [41]	2013	1 (1-3)	50%	12	10 (50%)	20%	2.5	0	2	1
Wang [45]	2014	1 (1-2)	85.7%	6	1 (14.3%)^‡^	28.6%	5 (NR)	0	1	0
Wang [8]	2015	NR	94.4%	12	1 (5.6%)	22.2%	5.5 (NR)	0	0	0
He [39]	2015	1.9	84%	12	15 (16%)	21%	4 (2-6)	0	1	4
Wang [46]	2016	NR	93%	12	NR	17%	5	0	1	1
Wang [43]	2017	NR	100%	12	0	0	—	0	2	1
Wang [26]	2018	1 (1-2)	86%	12	6 (20%)	14%	3 (2-8)	0	2	1
Yu [40]	2019	1.9	86%	60	11 (14%)	21%	NR	0	0	0

RFA: radiofrequency ablation; ESCN: esophageal squamous cell neoplasia; IQR: interquartile range; CR: complete remission; NR: not reported. ^†^No specific CR duration was defined. All patients had achieved CR during a follow-up of 10-28 month. ^‡^This patient was not eligible for further treatment for other severe clinical conditions.
